# Consolidating soil carbon turnover models by improved estimates of belowground carbon input

**DOI:** 10.1038/srep32568

**Published:** 2016-09-01

**Authors:** Arezoo Taghizadeh-Toosi, Bent T. Christensen, Margaret Glendining, Jørgen E. Olesen

**Affiliations:** 1 Aarhus University, Department of Agroecology, AU-Foulum, DK-8830 Tjele, Denmark; 2 Rothamsted Research, Department of Computational and Systems Biology, West Common, Harpenden, Herts AL5 2JQ, UK

## Abstract

World soil carbon (C) stocks are third only to those in the ocean and earth crust, and represent twice the amount currently present in the atmosphere. Therefore, any small change in the amount of soil organic C (SOC) may affect carbon dioxide (CO_2_) concentrations in the atmosphere. Dynamic models of SOC help reveal the interaction among soil carbon systems, climate and land management, and they are also frequently used to help assess SOC dynamics. Those models often use allometric functions to calculate soil C inputs in which the amount of C in both above and below ground crop residues are assumed to be proportional to crop harvest yield. Here we argue that simulating changes in SOC stocks based on C input that are proportional to crop yield is not supported by data from long-term experiments with measured SOC changes. Rather, there is evidence that root C inputs are largely independent of crop yield, but crop specific. We discuss implications of applying fixed belowground C input regardless of crop yield on agricultural greenhouse gas mitigation and accounting.

Soil carbon (C) is the third largest component of the global C cycle after the oceanic and the geologic C pools^1^,^2^. The amount of soil C (including peatlands, wetlands and permafrost areas) has been estimated at 2157–2293 Pg to a depth of 1 m, comprising 1462–1545 Pg in organic forms and 695–748 Pg as carbonate^1^. Soil organic matter is the source of soil organic carbon (SOC) and contains more organic carbon than global vegetation and the atmosphere combined^3^. In the soil, SOC is about twice the quantity of C presently in CO_2_ in the atmosphere^4^. Therefore, soils are globally considered the largest terrestrial ecosystem sink or source of atmospheric CO_2_ depending on land use and management practices. Globally, about 37% of land is used for agriculture and 10% of the land is under annual crops^5^. Over the last 150 years, agricultural soils have mostly been depleted in SOC due to cultivation and removal of crop residues, which suggests that agricultural land has potential to sequester SOC^6^,^7^. The accurate estimation of SOC content in agricultural lands and the factors that regulate SOC accumulation are critical aspects for evaluating the extent of this potential^4^. Numerous studies have attempted to quantify changes in SOC as a key component of global climate change, i.e. as affected by elevated CO_2_ or temperature using process-based models^8^,^9^. The formation of soil organic matter on agricultural land depends on the C input by above- and belowground crop residues including rhizodeposition during the growth period, C input from manures or other organic by-products, and the SOC stabilization mechanisms embedded in the simulation models^10^,^11^. Therefore, accurate estimations of the C input are fundamental for monitoring the development of SOC stocks of agricultural soils and also for modelling the potential for C sequestration and estimating changes in SOC content over several decades. However, reliable data on the C input of different crops is scarce and is often limited to rough estimates of above- and belowground C input using a plant C allocation approach which strongly relies on crop yield data and simplified C allocation coefficients^12^.

Many major soil C simulation models are built on the premise of the metabolic theory of ecology that a number of biological properties scale allometrically with body size according to a power-law over many orders of magnitude^13^. For that, total main harvestable crop products are normally used for calculating C mass in total net primary production (NPP) assuming a concentration of 0.45 g C g^−1^ dry matter in all crop parts and then calculating C biomass as a fraction of this value using biomass allocation functions^14^. A search of Web of Science in early 2016 for the combination of the words soil, carbon, model, allometric, NOT forest, NOT tree, NOT spectroscopy revealed 25 papers in the period 1990–2016. 19 of these papers use allometric functions for estimating soil C inputs. Many more studies were found when the name of specific SOC models were used instead of allometric and model keywords (e.g. ICBM (30 papers), CENTURY (107 papers), CN-SIM (18 papers), DAISY (30 papers)). Most of these studies were also explicitly or implicitly applying allometric functions for estimating soil C inputs. Such methods are also commonly and increasingly used for estimating changes in SOC as part of inventories for national accounting of greenhouse gases^15^. Other models use alternative approaches, for example RothC (118 papers) does not attempt to compute annual returns of plant C to the soil. Instead, the model is run ‘in reverse’ to generate monthly or yearly soil C inputs from known site, soil and weather data^16^,^17^.

Globally crop yields have increased greatly since the middle of the 20^th^ century, resulting from both an increase in the total biomass production (NPP) on agricultural land and a higher proportion of the biomass in harvestable material. For example, the ratio of the grain dry matter yield to total above ground biomass (harvest index, HI) in wheat has shown a mean increase from 0.35 in 1951–1955 to 0.45 in 1995–2010 due to general progress of plant breeding, a higher nutrient availability with intensive fertilisation, and improved control of weeds, pests and diseases^18^,^19^.

It has been suggested that increasing yields not only provide food for the growing population but also increase the sink of CO_2_ in agroecosystems due to the accumulation of SOC or reducing the decline rate of SOC^4^,^20^.

Among different crop parts which contribute to soil C input, below-ground residue inputs from roots and root exudates are of particular interest because they may contribute more to stable SOC pools than above-ground residues, partly because root derived C is more protected than other forms of C inputs due to physical protection of root materials by soil aggregates and fine pores^12^,^21^. However, estimating root C input is notoriously difficult because conventional root washing misses the fine roots and provides no estimates of the amount of C exuded from roots. Tracer techniques can be used to estimate C translocation by plants such as experimental labeling with ^13^C or ^14^C and determination of changes in ^13^C natural abundance. However, these techniques require more sophisticated equipment and measurement approaches. Furthermore, methods based on changes in the ^13^C natural abundance are restricted to systems with a change from C_3_ to C_4_ plants or vice versa^12^. Additionally, the estimations of total root C input from just one crop can differ due to the stage of growth of the plant, the environmental conditions, soil type, soil fertility and microbial activity^22^,^23^. Therefore, estimating below-ground C input using either measurements or allometric functional relationships remains a considerable challenge.

We hypothesize that a fixed relationship between above- and belowground biomass as used in allometric functions of most soil C turnover models overestimates belowground C inputs resulting from the technological progress that has increased crop yield. We examine the impact of nitrogen (N) fertilisation on soil C development using data from a long-term fertilisation experiment and suggest an improved method for estimating belowground soil C inputs in SOC models and in inventories of SOC change on agricultural land.

## Experimental Data

**Figure 1:**
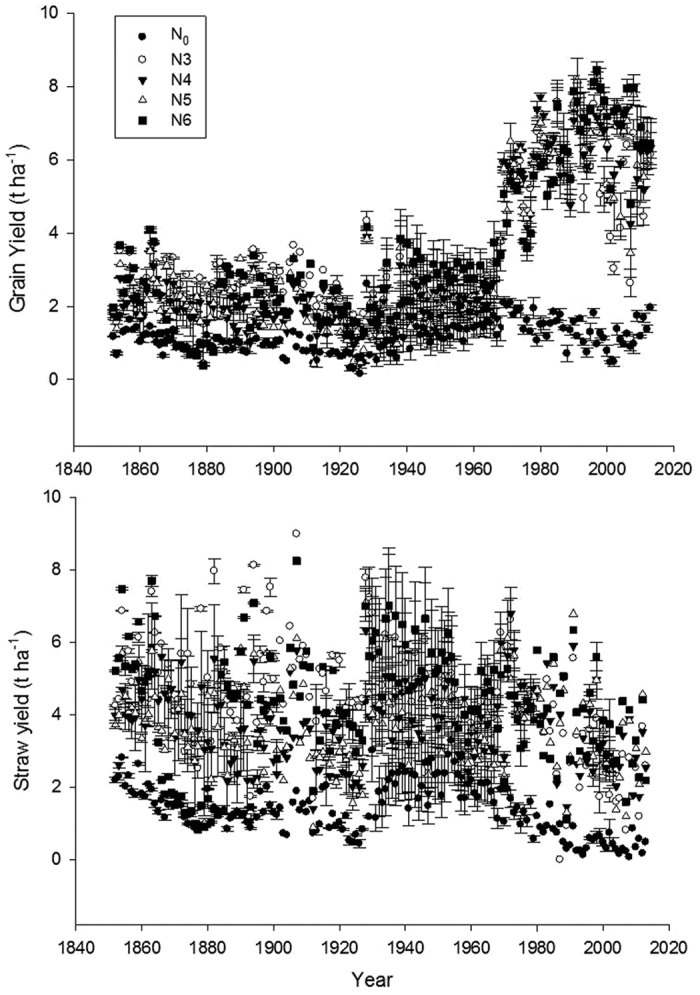
Trends in grain and straw yield (85% Dry Matter) in the Broadbalk wheat experiment, Rothamsted, UK (error bars = ± s.e.m., n = differs, see experimental data). See Table 1 for details of N treatments.

Long-term field experiments provide the best foundation for the experimental verification of SOC changes and for calibrating SOC turnover models. We use data from the Broadbalk Winter Wheat Experiment, a well-managed long-term field experiment which was established in 1843 at Rothamsted Research, Harpenden, Herts, UK, and has been under winter wheat (*Triticum aestivum* L.) and known management for over 170 years. For the first few years these treatments varied a little, but in 1852 a scheme of fertiliser treatments was established that has continued, with some modifications, until today. The site is thought to have been in arable cultivation since at least 1623, and probably much earlier^24^. The experiment does not have true, randomised replication, but has been divided into different sections over the course of the experiment^25^. From 1852 to 1925, the plots were divided into 2 halves; therefore n = 2 for mean and s.e.m. calculations (except 1902 to 1912 and 1914 to 1915 when n = 1). In 1926, the experiment was sub-divided in to five strips with generally one in fallow each year in sequence, to control weeds, crossing the treatment strips at right angles (n = 2 from 1926 to 1929, and n = 5 from 1930 to 1954). From 1955 to 1967, all plots were divided into seven strips, with one in fallow each year in sequence. The yields from the fallow plots were considered as zero. In 1968 the experiment was divided into 10 sections, in order to compare wheat grown in rotation with break crops with wheat grown continuously. For years 1968 to 2014 we have used the data from the three main continuous wheat sections (1, 6 and 9), excluding section 6 from 1968 to 1978 as it was still in a fallow rotation. The soil at Broadbalk is a well-drained clay loam to silty clay loam classified as an Aquic Paleudalf (USDA) and as a Chromic Luvisol^5^. Straw is removed at harvest each year, leaving above-ground residues of stubble, chaff and the uncollected straw. We selected the treatments from the continuous wheat sections, with a range of rates of N in mineral fertiliser (Table 1), resulting in different crop yields (Fig. 1). Austin *et al.* (1993) calculated HI based on total above-ground biomass (stubble, chaff and all of the straw) and compared an old long-strawed variety with a modern short-strawed variety, over three years and several different fertiliser treatments on Broadbalk^26^. The varieties were typical of those which historically have been grown on the experiment. The average HI was 0.30 for the old variety, and 0.47 for the modern variety^26^. Taking the period from 1852 to 1967 the ratio of grain yield to grain plus harvested straw yield was around 0.35 in Broadbalk. When winter wheat varieties were changed to modern short-straw varieties in 1968, the grain yield as proportion of grain plus harvested straw yield was changed substantially (less straw and more grain was produced). The average ratio of grain yield to grain plus straw yield for treatments N_3_, N_4_, N_5_, and N_6_ was 0.63 during the period 1968–2013, with no difference between the treatments. The mean for N_0_ was very similar to the other treatment, 0.65. Considering just the grain and harvested straw would underestimate the aboveground residues since stubble, chaff and unharvested straw was not measured. Nevertheless, it can be assumed that the ratio of grain to straw was constant, and allometric functions can be used for estimating aboveground residuals^27^. We used harvest index (HI: the ratio of grain to total aboveground biomass) to estimate above and belowground C input (see supplementary materials).

Measured SOC was calculated from % SOC and a standard soil weight of 2.88 × 10^6 ^kg ha^−1^ 0–23 cm (see supplementary materials for further details).

## Soil C model (C-TOOL)

For testing our hypothesis, we used a process based soil C model, C-TOOL^11^,^28^. In C-TOOL, the simulated SOC stock depends on the input of organic C from crops, its rate of decomposition, soil texture, initial soil C:N ratio and temperature. In the standard version of C-TOOL, allometric relations are used to derive the C inputs from measured crop yield (see supplementary materials). Here, C input from aboveground residues was calculated based on the estimated HI and measured grain and straw yields (Fig. 1) from 1852 to 2013. We have assumed HI = 0.35 from 1852 to 1970 and HI = 0.45 from 1980 to 2013. From 1970 to 1980, we assumed a linear increase of HI from 0.35 to 0.45. For root C input estimation, three approaches were pursued: **(1)** the C-TOOL allometric functions (see supplementary materials) were used to calculate the root C input of winter wheat into the soil for each treatment (hereafter called allometric root C input), **(2)** the N_3_ treatment was considered as a reference and the average N_3_ root C input (for 1852 to 2013) calculated from allometric functions was used as a constant root C input in all treatments (hereafter called fixed root C input), **(3)** the N_3_ treatment was considered as a reference and N_3_ root C input calculated from allometric functions for each year was applied in the corresponding year in the other treatments (hereafter called N_3_ root C input) from 1852 to 2013.

All treatments were assumed to have the same SOC content prior to the start of experiment. The model initial SOC content was estimated by minimising the sum of squares between simulated and estimated SOC in 1852 of N_0_ treatment using nonlinear curve–fitting function in MATLAB at the start of the model spin-up period (year 1822). That estimated initial SOC content was used for all treatments at the start of spin-up period (Spin-up period started in 1822). The spin-up period was 30 years and was used to initialise the model using similar C input calculated from allometric functions using winter wheat yield in 1852 for all treatments.

## Results

**Figure 2:**
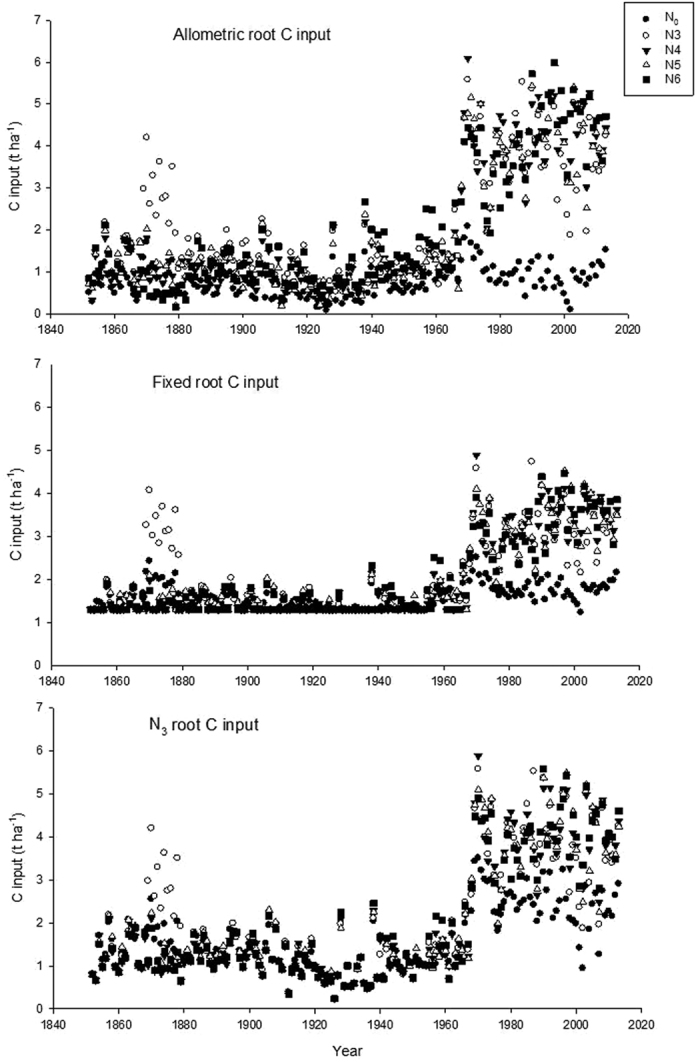
Trends in estimated total soil C inputs for different N fertiliser rate treatments of the Broadbalk winter wheat experiment at Rothamsted, UK. *Note:* different C calculations on each figure. See Table 1 for details of N treatments.

**Figure 3:**
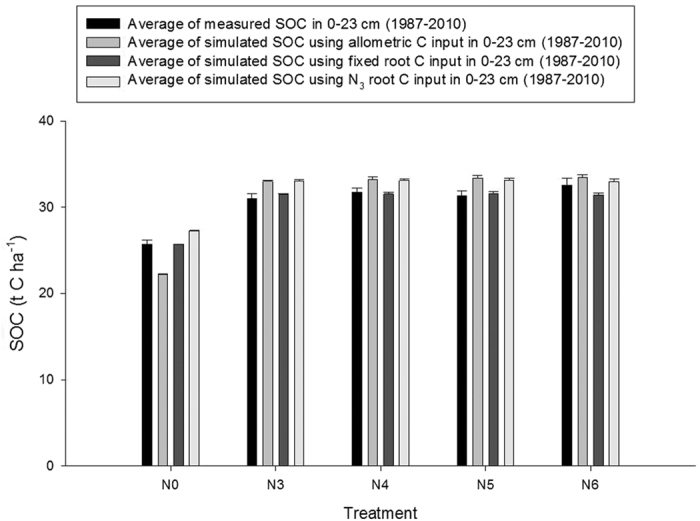
The average of SOC contents (measured SOC and simulated SOC using allometric root C input, fixed root C input and N_3_ root C input estimations for the period 1987–2010 (error bars = + s.e.m., n = 6 for measured values and n = 24 for simulated values). See Table 1 for details of N treatments.

Our study compared different methods for estimating soil C inputs using data from the long-term Broadbalk experiment at Rothamsted, UK, with different fertiliser rates providing different yields in winter wheat (Fig. 1). The wheat grain yields increased considerably over time in plots with the higher fertilisation rates while no increase in grain yield was observed in unfertilised plots. The estimated soil C input based on allometric root C input, fixed root C input or N_3_ root C input show markedly different estimates (Fig. 2). The average measured topsoil SOC contents in each treatment did not change significantly from year 1987 onwards (See supplementary materials, Fig. S4). Therefore, we used the average for 1987–2010 of measured SOC contents for comparing with the simulated values using different approaches for estimating root C input (Fig. 3).

## Discussion

The increasing wheat yields from 1960 to 1980 in all treatments receiving fertiliser was attributed to improvements in plant breeding and crop husbandry^29^. Crop breeding, particularly cereals, has resulted in the preferential allocation of assimilated C to grains (higher HI)^30^. However, total biomass production has also been increasing, due to a combination of improved crop varieties, better plant nutrition and improved crop protection. Increased N fertiliser rates result in increased yields; however, this relationship is non-linear with a saturation response at higher N rates (Fig. 1). The carbon input as a proportion of winter wheat yield varies in literature, but it was considered 86% by Jenkinson and Rayner (1977)^31^. Chrinida *et al.* (2012) measured grain yield (9.5 t ha^−1^), the amounts of shoot C (5.1 t C ha^−1^) and root C (0.9 t C ha^−1^) of winter wheat in the inorganic fertilised based rotation in Denmark^32^.

In SOC models that use allometric functions to estimate C inputs, higher grain yields produced by higher N fertilisation rates will result in enhanced above- and belowground biomass and thereby greater inputs of C in crop residues (stubble and roots) to the soil. As a result, higher SOC contents would be expected in high compared to low-yielding cropping systems. For the treatment with no N fertilisation (N_0_), SOC was measured to be lower compared with the treatments with higher N rates. When N fertiliser was applied at 144 kg N ha^−1^ yr^−1^ (reference rate, N_3,_ since 1852), higher SOC contents were as expected obtained compared with no N application. However, rates of N fertiliser above the reference rate (N_4_, N_5_ and N_6_) did not increase topsoil SOC contents much more, although these treatments had only been applied since 1968 (N_4_) and 1985 (N_5_ and N_6_). In contrast, the simulations of SOC contents using the allometric function for calculating C input showed higher SOC contents in topsoil of N fertilised treatments than measured topsoil organic C contents (ranged from extra 1 to 4 t C accumulation ha^−1^, see supplementary materials, Figure S1). We surmise that the deviation between measured and simulated SOC contents with different level of N fertiliser can be ascribed mainly to overestimation of belowground C inputs. Higher N fertiliser rates do not necessarily increase belowground biomass, possibly due to a relative decrease in cumulative fine root production as a result of fertilisation^32^. Root biomass has shown less response to added N than aboveground biomass causing the below- to aboveground ratio to decline with increasing N rates^33^. When we used N_3_ root C input for each year in different treatments, the simulated SOC contents were slightly overestimated by 0.4 to 2 t C ha^−1^ in N_4_, N_5_ and N_6_ treatments (see supplementary materials, Figure S2). This means that there is a need to estimate the belowground C input using an alternative to the allometric function, which could be independently derived, reliable belowground C input data. In our simulations, using fixed root C input resulted in a closer match between simulated and measured topsoil SOC (difference; 0.02 to 1 t C ha^−1^, see supplementary materials, Figure S3). There was a slight difference in SOC contents between measured and simulated using fixed root C input in N_3_ (reference N rate, Fig. 3). We believe that difference might be due to slight overestimation of aboveground C input from winter wheat residues and stubbles, possibly due to changes in the HI not accounted for in our assumptions^34^. These results indicate that estimation of changes in SOC on arable land using allometric functions probably overestimate the ability of the land surface to accumulate C from technological progress. These findings emphasize the need to better quantify and represent shifting plant C allocation in the face of changing technologies that lead to yield improvements, but not to equivalent increases in root C inputs.

## Conclusion

Use of crop yields and application of strict allometric functions for estimating C inputs from belowground crop residues and exudates in carbon models can lead to erroneous estimates of belowground C inputs in agricultural systems. The commonly applied method of assuming a fixed ratio between crop yield and root C inputs will tend to overestimate root C inputs for technologies that improve harvestable yield. However, using a process based model like C-TOOL and allometric functions in combination with fixed belowground C input value estimated from standard crop management may result in more accurate estimation of SOC trends in agricultural soils. Such an approach would contribute to an improved assessment of soil C sequestration potentials, in particular when considering measures that enhance harvestable crop yield.

## Additional Information

**How to cite this article**: Taghizadeh-Toosi, A. *et al.* Consolidating soil carbon turnover models by improved estimates of belowground carbon input. *Sci. Rep.* **6**, 32568; doi: 10.1038/srep32568 (2016).

## Supplementary Material

Supplementary Information

## Figures and Tables

**Table 1:** Treatments with continuous winter wheat in the Broadbalk experiment at Rothamsted, UK, used in the current study.

Treatment	N rate (kg N ha^−1^) 1852–1985	N rate (kg N ha^−1^) 1985-onwards
N_0_	0	0
N_3_	144	144
N_4_	48 (year 1852–1967) & 192 (year 1968–1984)	192
N_5_	96 (year 1852–1967) & 144 (year 1968–1984)	240
N_6_	192 (year 1852–64), 0 (year 1865–83), 96 (year 1884–1984)	288

All plots also receive non-limiting P, K, Na and Mg.
