# Supplementary material for: Consolidating soil carbon turnover models by improved estimates of belowground carbon input
Source: Sci Rep. 2016 Sep 1;6:32568. doi: 10.1038/srep32568 (PMC5007648; doi:10.1038/srep32568)
Supplement: Supplementary Information [file srep32568-s1.pdf]

Consolidating soil carbon turnover models by improved estimates of belowground carbon input

Arezoo Taghizadeh-Toosi[1,*], Bent T. Christensen[1], Margaret Glendining[2] & Jørgen E. Olesen[1]

[1]Aarhus University, Department of Agroecology, AU-Foulum, DK-8830 Tjele, Denmark
[2]Rothamsted Research, Department of Computational and Systems Biology, West Common, Harpenden, Herts AL5 2JQ, UK
*Corresponding author: Arezoo.Taghizadeh-Toosi@agro.au.dk

Supplementary online material

C-TOOL allometric equation

Allometric equations to calculate C inputs into the soil is described in Taghizadeh-Toosi *et al.* (2014) as part of the C-TOOL model and Keel *et al.* (2016). The amount of C (C_P) in dry matter yield (Y_{DM}) of the grain and straw is calculated as Equation 1, CC is a C concentration of 0.45 g g^{-1} in all crop parts:

$$C_P = Y_{DM}\, CC \qquad \text{Equation 1}$$

The aboveground carbon in crop residues (C_S) depends on winter wheat harvest index (HI = the ratio of grain to total aboveground biomass = 0.45) and is calculated as:

$$C_S = C_P/HI \qquad \text{Equation 2}$$

The amount of aboveground residues left after harvesting grain and straw (C_L) is calculated as the difference between C_s and C_p (grain & straw).

$$C_L=C_s-C_{p(grain)}-C_{p(straw)}$$

Belowground C input from roots and rhizodeposition C_{iRE} is calculated as:

$$C_{iRE} = Cs/((1- F_{RE})\text{-Cs}) \qquad \text{Equation 3}$$

where F_{RE} root and rhizodeposition C (belowground C) as proportion of total C assimilation (F_{RE} = 0.25 for winter wheat).

The C-TOOL model has a topsoil (0-25cm) and a subsoil layer (25-100cm) to which C inputs are distributed as follows:

$$C_{top} = C_L + F_{top}\, C_{iRE} \qquad \text{Equation 4}$$

$$C_{sub} = (1- F_{top})\, C_{iRE} \qquad \text{Equation 5}$$

F_{top} is the proportion of belowground C deposited in the topsoil and is set to 0.7 for winter wheat.

Measurement of SOC in the Broadbalk experiment

SOC was calculated from measured % SOC and a standard soil weight of 2.88 x 10^6 kg ha^{-1} 0-23cm. The 1843 value was estimated, and the 1865 value calculated from total soil N content in 1865 and C:N ratio in 1893. SOC measurements started in 1881, as total soil C minus $CaCO_3$-C, with analysis of air-dried finely ground (0.354mm) soil. Samples were taken from the whole experiment up to 1966, then from the continuous wheat sections (sections 1, 6 and 9) only. Samples from 1881, 1893, 1914, 1936 and 1944 were re-analysed in 2001 – 4 for total C by combustion (LECO) and for $CaCO_3$-C by manometry; this method was also used for samples from 1992 onwards. For further details, refer to http://www.era.rothamsted.ac.uk/ Broadbalk Soil Organic Carbon Open Access data.

References

Taghizadeh-Toosi, A. *et al.* C-TOOL: A simple model for simulating whole-profile carbon storage in temperate agricultural soils. *Ecological Modelling* **292**, 11-25 (2014b).

Keel, S. G. *et al.* Large uncertainty in soil carbon modelling related to carbon input calculation method. Accepted in *European Journal of Soil Science* (2016).

Table S1. Average measured and simulated SOC contents in 0 – 23 cm using three methods of root C input calculations from 1987 to 2010.

	N_0	N_3	N_4	N_5	N_6
Average measured SOC contents 1987-2010 (t C ha^{-1})	25.73	31.01	31.78	31.30	32.59
Average simulated SOC (t C ha^{-1}) contents 1987-2010 Allometric root C calculations	22.22	33.02	33.25	33.38	33.43
Average simulated SOC (t C ha^{-1}) contents 1987-2010 N_3 root C input calculations	27.27	33.04	33.10	33.16	33.00
Average simulated SOC (t C ha^{-1}) contents 1987-2010 Fixed root C calculations	25.71	31.48	31.54	31.60	31.44

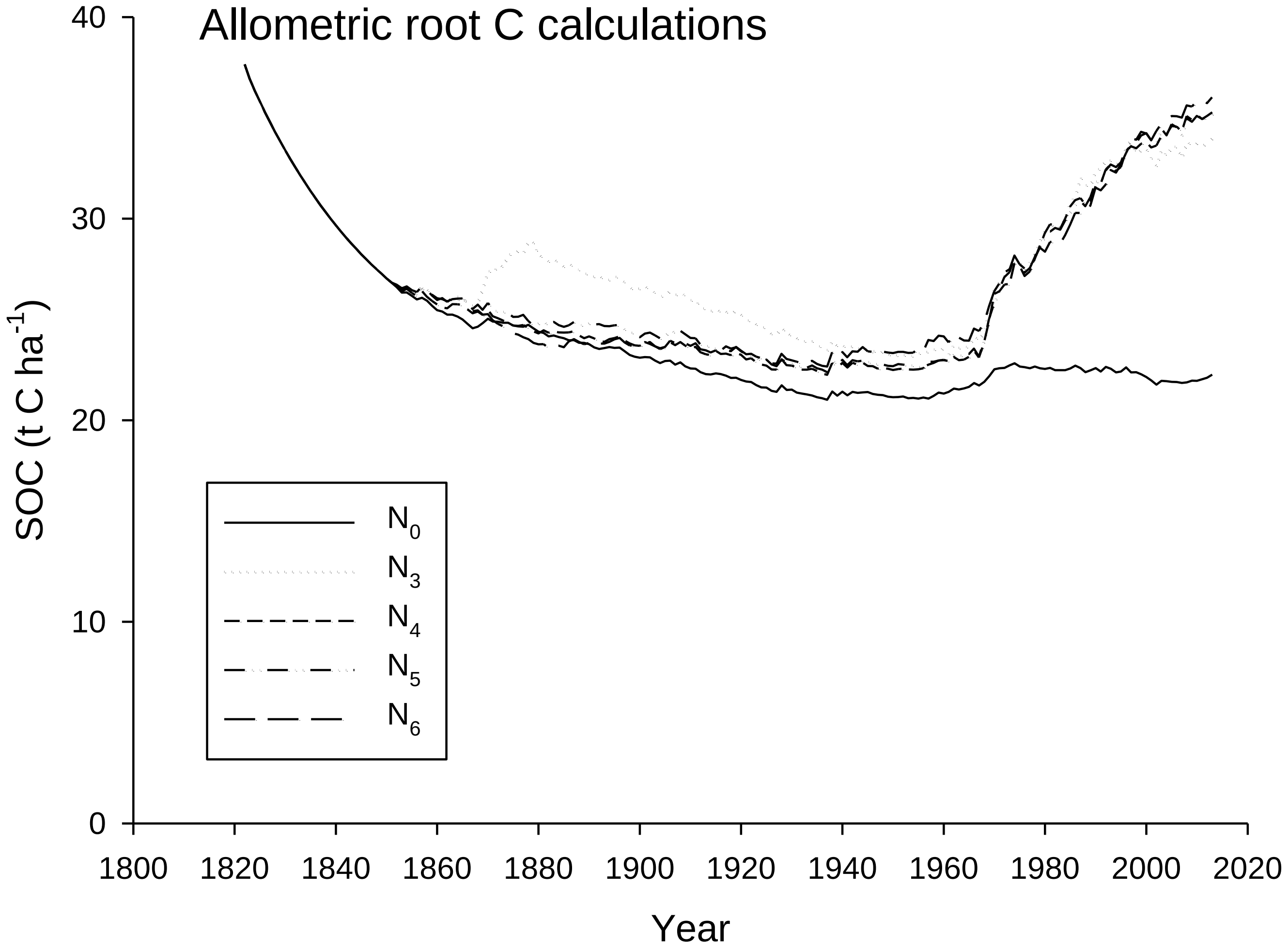

Figure S1. Simulated soil organic carbon changes in 0 – 23 cm of the Broadbalk winter wheat experiment at Rothamsted, UK, allometric functions were used to calculate C input. See Table 1 for details of N treatments.

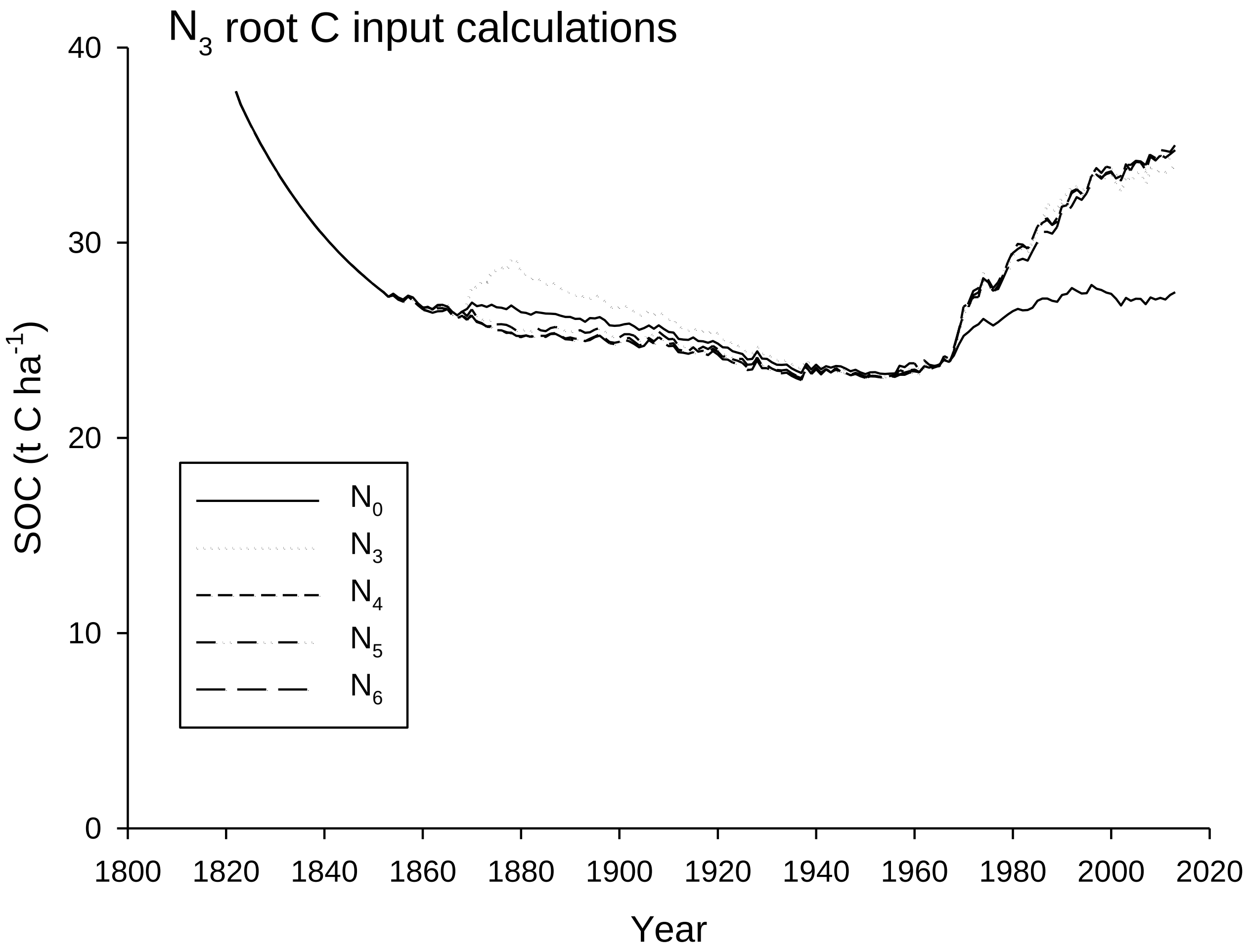

Figure S2. Simulated soil organic carbon changes in 0 – 23 cm of the Broadbalk winter wheat experiment at Rothamsted, UK, N_3 root C input calculations were used. See Table 1 for details of N treatments.

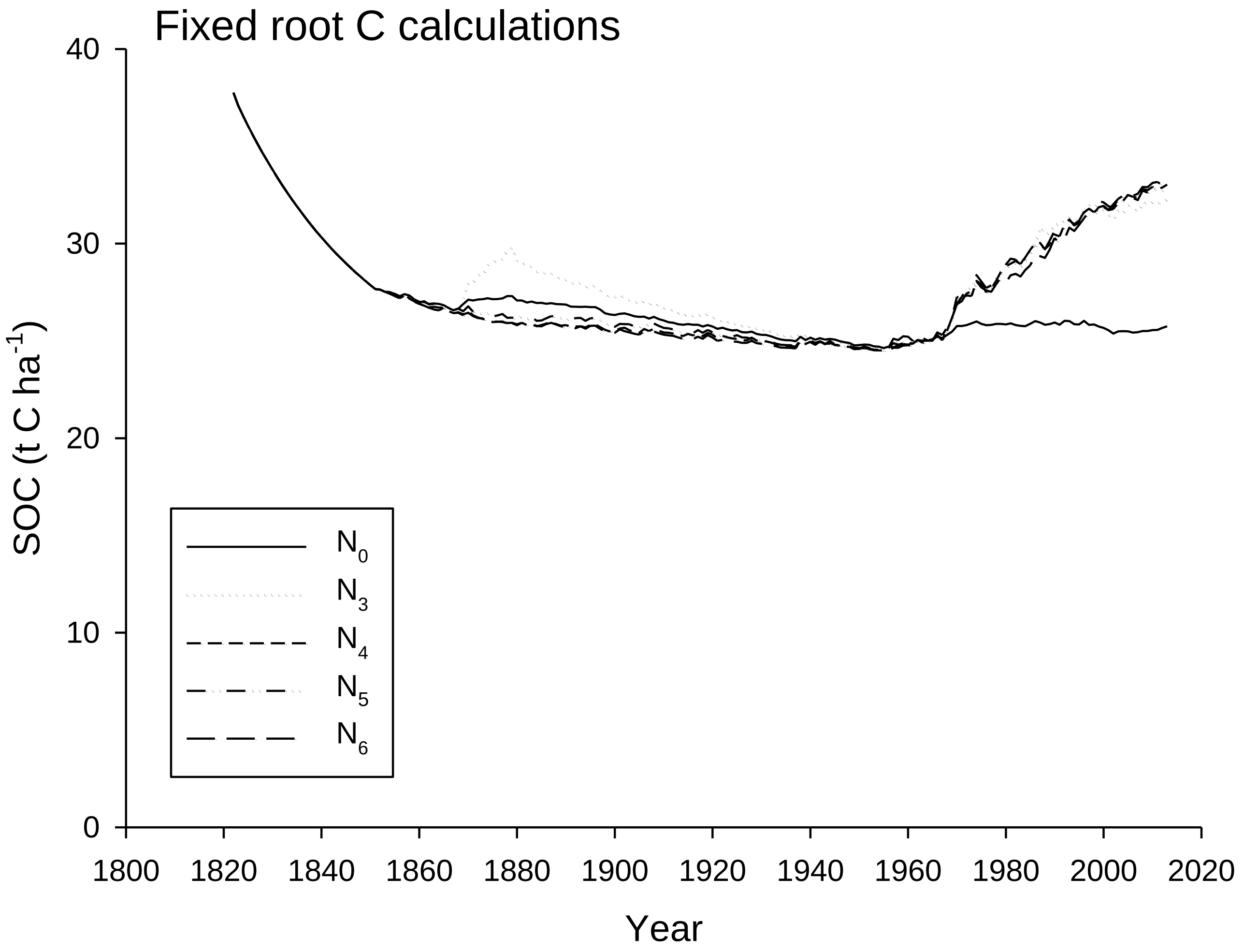

Figure S3. Simulated soil organic carbon changes in 0 – 23 cm of the Broadbalk winter wheat experiment at Rothamsted, UK, fixed root C input calculations were used. See Table 1 for details of N treatments.

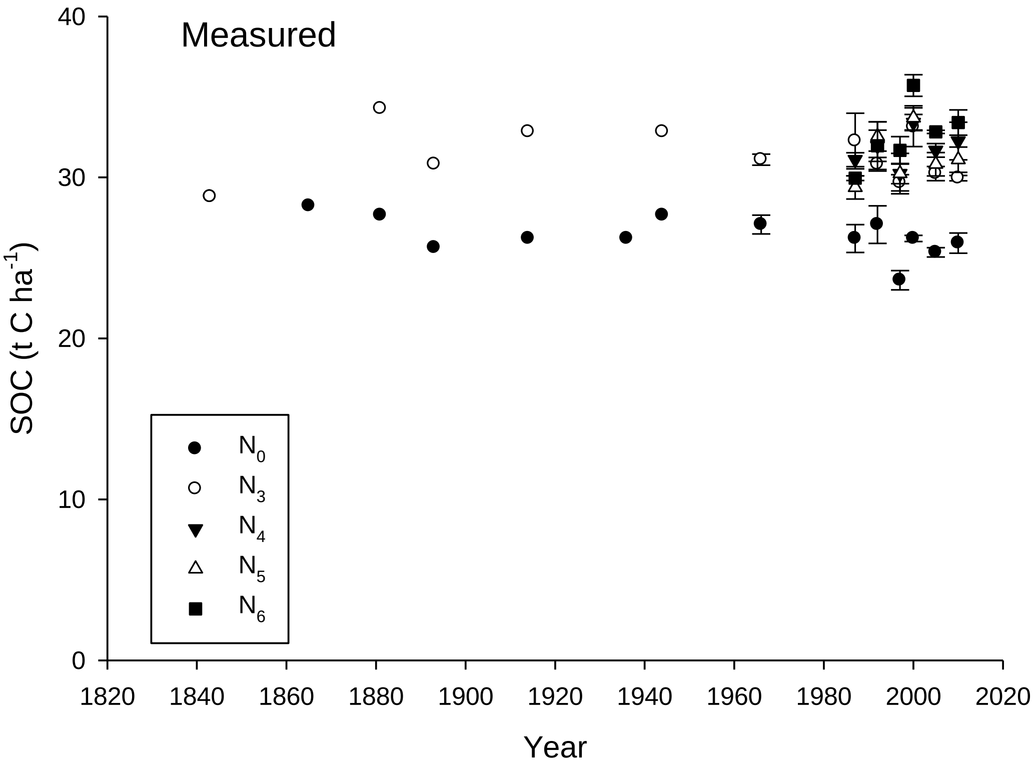

Figure S4. Measured soil organic carbon in 0 – 23 cm of the Broadbalk winter wheat experiment at Rothamsted, UK. 1843 value estimated, 1865 values calculated from total soil % N and soil C:N in 1893. The other values calculated from % SOC and a standard soil weight of 2.88 × 10^6 kg ha^{-1}. Whole experiment up to 1966, then continuous wheat sections (sections 1, 6 and 9) only (error bars = ± s.e.m., n = 2 for year 1987 and n =3 for year 1992, 1997, 2000, 2005 and 2010). See Table 1 for details of N treatments.
